# Developing the Standard of Care for Post-Concussion Treatment: Neuroimaging-Guided Rehabilitation of Neurovascular Coupling

**DOI:** 10.2174/1874440001711010058

**Published:** 2017-10-24

**Authors:** Benjamin H. Wing, Braden J. Tucker, Alina K. Fong, Mark D. Allen

**Affiliations:** 1 Cognitive FX, Provo, UT, USA; 2 Notus Neuropsychological Imaging, Orem, UT, USA; 3 Utah Valley Regional Medical Center, Provo, UT, USA; 4 Case Western Reserve University School of Medicine, Cleveland, OH, USA; 5 American University of the Caribbean School of Medicine, Cupecoy, St. Maarten, USA

**Keywords:** Mild Traumatic Brain Injury (mTBI), Concussion, Post-Concussion Syndrome (PCS), Neurovascular Coupling (NVC), Neurovascular Uncoupling (NVU), Neurorehabilitation, Blood Oxygen Level Dependent (BOLD) signals, Functional neurocognitive imaging^TM^ (fNCI)

## Abstract

**Background:**

Emerging research proposes the imbalance between microvascular supply and metabolic demand as a contributing factor in the pathophysiology of mild traumatic brain injury. Prolonged effects on the dysregulation of neurovascular coupling may explain persistent symptomatic models such as Post-Concussion Syndrome.

**Objective:**

Increased knowledge of what we refer to as neurovascular uncoupling provides a template for establishing a new concussion treatment standard in the assessment and therapeutic guidance of concussion.

**Methods:**

The degree and localization of neurovascular uncoupling were statistically contextualized against a normative-based atlas in 270 concussed patients. Functional NeuroCognitive Imaging^TM^ was used to establish pre-treatment benchmarks and guide neurotherapy. Conventional and functional neurocognitive imaging-directed measures were used to evaluate post-rehabilitative outcomes.

**Results:**

Functional neurocognitive imaging was successful in identifying regions of Neurovascular uncoupling unique to each patient’s brain and concussion profile. Longitudinal objective outcome measures demonstrated timely and lasting improvement of neurovascular coupling functioning in a significant majority of patients.

**Conclusion:**

We present practice-based evidence supporting the clinical administration of functional neurocognitive imaging with particular efficacy in the neurorehabilitation of concussion. We advocate the reliability of functional neurocognitive imaging in assessing severity and localization of neurovascular uncoupling, and promote its use in the therapeutic guidance and neurorehabilitation of mild traumatic brain injury. We further support the continual exploration of other potential pathophysiological alterations resulting from concussion.

## 1 INTRODUCTION

### 1.1 Mild Traumatic Brain Injury

Improved awareness of the epidemiology, etiology, presentation, and sequela of mild traumatic brain injury (mTBI) coupled with trends toward more aggressive play in sports has led to significant increases in its prevalence and diagnosis [1, 2]. Consequently, increased health care demand has led to an urgent need for valid guidelines to accurately assess and diagnose this complex injury [3]. Recently, extensive efforts have been made to understand the diagnosis, prognosis, and treatment of mTBI, as well as the persistent underlying physiological changes that may occur in post-concussion syndrome (PCS), such as neurovascular signaling disruptions that take place within cortical micro-structure. However, most current treatment approaches do not incorporate advanced scientific findings of the underlying causes of persistent mTBI dysfunction into rehabilitative practice, but rather rely on traditional strategies that are largely focused on reducing overt symptoms.

Current guidelines for treatment of acute mTBI center on limiting physical and cognitive activity while reducing exposure to symptom-aggravating stimuli [2]. For more chronic symptoms, the standing recommendation for treatment of post-concussion conditions is a symptoms-based approach [1]. Although the current treatment methods promote patient recovery from some mTBI symptoms, this approach may fail to address the underlying etiology and pathophysiology of concussions resulting in longitudinal alterations in cognitive, occupational, emotional, and neuromuscular function [4-7]. Consequently, a standard for care that addresses and treats the underlying pathology is needed for effective treatment of post-concussion symptoms.

### 1.2 Neurovascular Coupling

Recent scientific investigations on the effects of concussion suggest that disruptions to neuronally-triggered regional cerebral blood flow (rCBF) may account for a significant portion of PCS symptoms [8, 9]. Anything less than precise neurovascular coupling (NVC) between neural activity and blood flow may result in obvious cognitive and physical deficits [10, 11]. Although permanent gross structural brain damage is not observed in concussion/mTBI (by definition), there is clear evidence for long-term disruption of NVC, associated with measurable neurocognitive dysfunction typical in PCS symptomology [12-16]. We refer to this phenomenon as Neurovascular Uncoupling (NVU).

Given the key role that NVU is likely to play in PCS, effective treatment should include 1) an objective neuroimaging method for detecting NVU in the brain, and 2) an informed rehabilitation protocol with advanced techniques for restoring normal NVC. We present here data from a large sample of mTBI patients assessing the effectiveness of one specific treatment program, Enhanced Performance in Cognition^TM^ (EPIC) treatment, which employs a novel, evidence-based system of atypically aggressive NVC recovery therapies, and advocate its methodologies for developing new, pathologically-based standards of care for post-concussion treatment.

## 2 MATERIALS AND METHODS

### 2.1 Functional Magnetic Resonance Imaging & NVC

Functional magnetic resonance imaging (fMRI) has been used extensively over the last two decades as the dominant research tool for understanding human brain function. fMRI is often described as a method that detects ‘brain activity.’ However, a more accurate description of fMRI would be that it detects efficiency in NVC. Although fMRI does not directly measure neural activation, it has nonetheless proven to be a highly reliable tool in brain function mapping, given the precise coupling that normally exists in the *healthy* brain between neural activity and the blood oxygen level dependent (BOLD) signal that is detected by fMRI [17]. However, due to the very fact that fMRI is a more direct measure of NVC efficiency than neural activity itself, it is currently the most ideal imaging tool to measure dissociations between NVC and what is otherwise normal brain cell functioning in *unhealthy* brains, specifically as found in PCS.

Given the special nature of fMRI with regard to mTBI dysfunction, many high-quality research studies over the last decade have used fMRI to investigate neurocognitive effects of concussion [18-21], and, to a limited extent, treatment outcomes [22, 23]. However, the fMRI method as standardly employed in scientific research lacks essential features necessary for clinical assessment: 1) A concurrently validated, reliable, and objective standardized protocol appropriate for the scanning environment, and 2) A clinically acceptable normative-based contextualization procedure for appropriate individualized patient assessment [8].

#### 2.1.1 Standardized Protocol: Functional Task Battery

Our unique Functional NeuroCognitive Imaging^TM^ (fNCI) assessment protocol combines the validity of conventional neuropsychologic testing standards with the reliability and objectivity of informational data output provided by fMRI. The testing battery underwent iterative pilot testing to ensure concurrent validity, reliability, objectivity, and suitability for the scanning environment [24, 25].

The functional task battery, *Notus NeuroCogs*^TM^, is comprised of six neuropsychologic test adaptations: the functional Matrix Reasoning Test^TM^ (*f-MRT*), the functional Trail Making Test-B^TM^ (*f-TMTB*), the functional Picture Naming Test^TM^ (*f-PNT*), the functional Face Memory Test^TM^ (*f-FMT*), the functional Verbal Memory Test^TM^ (*f-VMT*), and the functional Verbal Fluency Test^TM^ (*f-VFT*). Each of the six tasks includes eight Test Phases presented in alternating fashion with Rest Phases, in which the subject is asked to silently count from 1 to 10. Compliance monitoring is performed at intervals during each task. Operative descriptions are outlined in Table **1** below:

#### 2.1.2 Standardized Protocol: Imaging Parameters

All scanning was performed at the same location using the same standardized administration protocol and functional task battery to limit platform-generated variability.

Functional images were acquired with a 1.5-T GE scanner using an EPIBOLD sequence with the critical parameters TR = 2000 ms; TE = 40 ms; Flip Angle = 90. Images were acquired at 23 contiguous axial locations with a slice thickness of 5 mm, 0 mm interslice gap, with a 3.75 x 3.75 mm in-plane resolution and a 64 x 64 matrix of individual sample points, producing a total of 64 x 64 x 23 voxels for entire brain coverage. Preprocessing procedures included acquisition time realignment, using sinc interpolation, followed by motion correction with echo-planar imaging (EPI) distortion unwarping. No head movement exceeded 1 mm translation or 1° rotation displacement. Images were spatially smoothed with an 8-mm FWHM Gaussian kernel. A high-resolution 3D SPGR was coregistered to each individual’s mean functional image in order to perform subject-specific functional region analyses that take into account individual variability in cortical landmark organization, for the purposes individual activation extraction requisite for normative reference atlas construction described below.

For each test condition, a time-series analysis of covariance (ANCOVA) implemented in SPM8 was used to test each voxel, for each subject, against the null-hypothesis that changes in BOLD signal in that voxel, over the duration of the experiment, did not significantly correlate with the temporal sequencing of the cognitive task of interest. A boxcar waveform convolved with a synthetic Hemodynamic Response Function (HRF) with a 4-s lag-to-peak was used to model task-related activation. The data were high-passed-filtered in time, using a set of discrete cosine basis functions with a cut-off period of 128 s, and conditioned for temporal autocorrelations using AR1 correction.

#### 2.1.3 Normative-Based Contextualization: Statistical Methods

In order to further support the reliability of our testing battery and employ its clinical application, we utilized a group-summary analysis approach, where single-subject NVC is compared against normative datasets.

Methods for deriving normative fMRI data follow a two-stage process [24-26]. First, statistical activation maps were computed for each subject (as described above), and anatomical boundaries were identified for each subject by a neuroanatomical expert. Second, activation peaks were identified according to an objective algorithm within pre-specified anatomical regions (as described in the next paragraph). These pre-specified regions were determined based on prior group-averaging studies using the similar task-related experimental protocols. Importantly, the normative database in this study is not a result of standard group-averaging techniques typically used in fMRI research. Instead, it is composed of individual data points extracted from individual brain analyses, both in terms of anatomy and activation. This allows the protocols to be acceptably applied at the single-subject level.

The procedure for designating and identifying anatomical region boundaries essentially follows the automated anatomical labeling scheme described by Tzourio-Mazoyer and colleagues [27]. However, it is important to note that as the database was not derived via group averaging, no automated (probabilistic) segmentation was performed. Likewise, no brain space normalization was performed, as is prerequisite for group-averaging procedures. Rather, for this database process, region boundaries were determined in a precise manner for each subject’s non-normalized brain. However, each anatomical image was coregistered to the subject’s corresponding mean functional image map. Each activation map was also smoothed with a 1.5 mm FWHM Gaussian spatial filter, in order to condition extreme *t*-value spikes within peak clusters.

After anatomical parcellation, individual functional activation maps (with a single *t*-value assigned to each voxel) were overlain for identification of region peaks. Regions were then inspected for cluster peaks, following these guidelines: If the maximum value within a region belonged to a cluster with a centroid in an adjacent region (i.e., the highest intensity voxel fell at the border of an adjacent region), the region was determined to not have a peak. When more than one peak was identified in a region, the locations of the peaks were catalogued and, if consistently found across subjects (>30%), used to motive further functional region boundary divisions for the cognitive task protocol being analyzed. However, only those regions (or subregions) with peak clusters present in at least 70% of control subjects were included in the normative data set for each protocol (although in most regions, agreement across subjects in cluster presence/absence exceeded 90%). Following *t*-value extraction from each subject, means and standard deviations were computed for each region and used to derive normalized *z*-scores. For each of the six protocols, 8-12 regions met the above requirements for inclusion in the database, for a total of 57 regions. The distributions of *t*-values in each anatomical region were assessed for normality prior to *z*-score transformation, on the basis of 59 independent control subjects. Normative reference demographics are outlined in Table **2** below. Anderson–Darling sample-size-adjusted tests for normality determined the distributions of each of the 57 regions to be sufficiently normal, with estimates ranging from moderate (A2* = 0.59, p = 0.11) to high (A2* = 0.18, p = 0.91).

#### 2.1.4 Normative-Based Contextualization: Patient Demographics

These distributive properties formulated a three-dimensional activation standard, or normative atlas, which was later used to statistically contextualize both severity and localization of NVU in 270 concussed patients. Patient demographics can be visualized in Table **3** below.

### 2.2 Rehabilitative Measurements

The fNCI Severity Index Score (SIS) was developed in order to represent the overall presence of PCS biomarkers in an individual with a single summary score. The score is computed by taking the average activation deviation (*z*-score) across all target regions associated with a given biomarker within an individual and multiplying it by the positive predictive value for that biomarker. The SIS, then is the sum of this computation for all 5 biomarkers. The SIS was found to have an approximate range of 3-8, for PCS patients. For healthy control subjects, the PCS range was approximately 1-4 (scores below 1 are nearly impossible with realistic brain activation variability), with a mean of 2.04. As the SIS is intended to be used primarily with patients who are independently diagnosed with probable PCS, as opposed to healthy controls, a patient-based SIS scale was developed (the one reported in this study) in which the value 0 was set to the healthy control mean, such that PCS scores tend to fall within the range 0-6. Furthermore, the standard deviation from the healthy controls (0.85) was used to establish approximate severity range labels (e.g., mild, moderate, severe) for convenience in communicating results with patients and health care practitioners.

SIS is a summary computation of the degree of deviation from normal activation levels observed in an individual. Given that the BOLD signal is a measure of intact NVC [28], the SIS essentially provides a very precise evaluation of NVC functioning. Thus, SIS directly represents NVU.

**Fig. (1):**
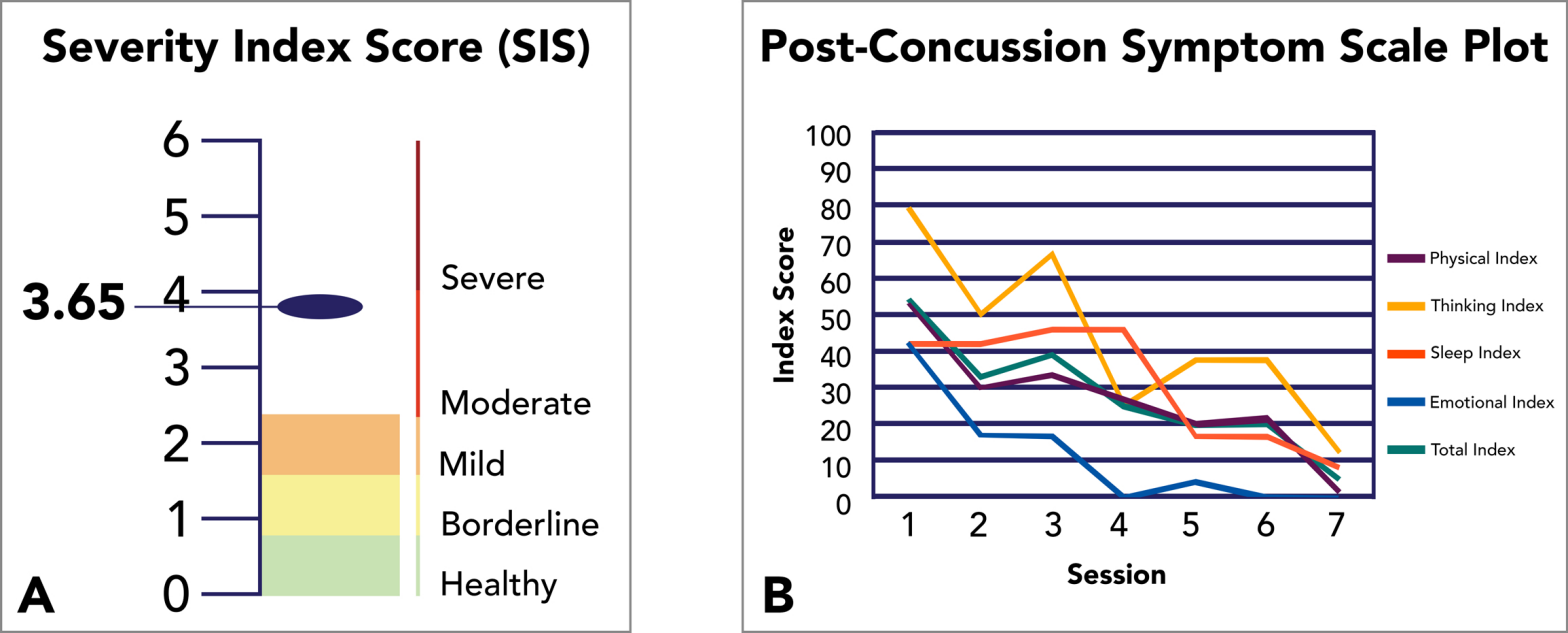
Method for Visualizing SIS and PCSS Scores Before, During, and After Treatment. Severity Index Scores (A) and Post-Concussion Symptom Scales (B) are graphically displayed for clinical purposes as shown above. A) The SIS score for an individual patient is displayed in the context of Healthy, Borderline, Mild, Moderate, and Severe ranges of PCS biomarker presence. B) Symptom subscales and total index scores are displayed for each day during treatment to track patient progress.

Pre-EPIC SIS were computed to customize therapeutic priorities based on patient need, and provided baselines with which to objectively measure therapeutic effectiveness, post-treatment. Post-EPIC evaluations were carried out upon completion of individualized rehabilitation routines averaging 4.1 days between scans. In addition, these evaluations utilized different versions of *Notus NeuroCogs* to limit practice effects. In addition to the objective SIS measurement, we retain the use of Post-Concussion Symptom Scales (PCSS)—an existing neurocognitive assessment standard in the form of a self-report survey completed before, during, and after treatment—to supplement our measurement of the effectiveness of fNCI-directed treatment (Fig. **1**).

### 2.3 EPIC Treatment

In addition to standardized test administration, normative-based assessment, and individual, image-guided measurements, our EPIC treatment exploits the latest knowledge of NVC and its role in PCS by integrating three fundamental rehabilitation facets for each patient: Prepare, Activate, Rest. Specific therapeutic activities included in each of these three phases of EPIC treatment are the product of research, clinical experience, screening, and empirical testing [29-34]. EPIC therapy implements a cyclical pattern of treatment where patients are rotated through therapies and activities that promote cognitive and physical rehabilitation from concussions. The preparatory stage of therapy includes varying degrees of aerobic exercise and neuromuscular therapy, based on patient capacity, to enhance cerebral blood flow in anticipation of cognitive therapy while also encouraging neurosensory rehabilitation [32, 33]. This phase of therapy lasts 50 minutes in duration and is followed by the activation portion of EPIC. We implement traditional cognitive and occupational therapy techniques for a subsequent 50 minutes of tailored treatment to target specific deficits for each patient. Complex, multistep problem solving, logic puzzles, functional and short-term memory challenges, digital therapeutic games, visual exercises, motor skill retraining, and psychosocial therapy are all implemented in this stage of treatment [30]. Additionally, Dynavision^TM^ is incorporated into therapy as a means of treating visuospatial deficits and to enhance multitasking and cognitive processing. A destimulation period of varying duration follows to allow for recovery before another round of therapy will begin [34]. Brainwave entrainment destimulation is used promote neural wave harmonization and reduce cognitive activity [29]. This cycle is repeated for 6-8 hours a day over a typical 4-day treatment period that can be adjusted based upon symptom severity and resolution.

These therapeutic approaches address a wide range of cognitive, sensory and physical functions. The key to success, however, is not necessarily the therapies themselves, but the manner in which the therapies are applied (i.e., timing and order). For example, a carefully guided rotation between physical/cardio challenge and cognitive or sensory processing challenge is a central feature in restoring the timed pairing between rCBF and regional neural firing. Our multidisciplinary team was successful in restoring pre-traumatic NVC through the cyclical application of these rehabilitative principles supplied in tandem with the benefit of appropriately timed neurocognitive exercise [35, 36].

A multi-method approach was also employed to avoid overexertion of brain regions with severe NVU. Accordingly, cyclical brain training aimed to strengthen surrounding neural correlate systems, which, in turn, support the more severely uncoupled regions. Sustained therapy over a multi-day period was shown to promote accelerated and longitudinal restoration of pre-traumatic NVC.

## 3 RESULTS

### 3.1 Patient Outcomes

**Fig. (2):**
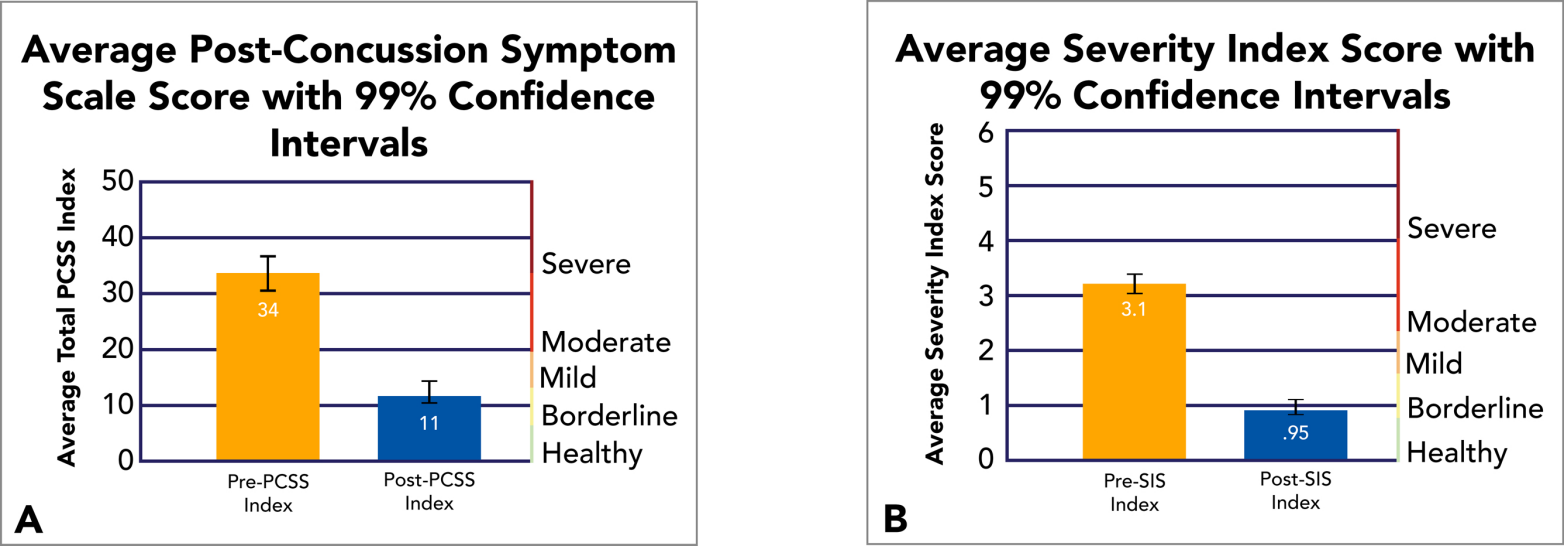
Average SIS and PCSS Pre- and Post-EPIC Treatment. Average pre- and post-treatment scores are reported for PCSS outcomes (A) and fNCI-based SIS outcomes (B).

Data collection and analysis confirmed the effectiveness of fNCI-directed treatment in accelerated concussion rehabilitation. Quantitative improvement was observed on both objective fNCI measurements and subjective symptom report measurements after an average of 4.1 days of EPIC treatment. Average pre- and post-treatment measurements are reported for a sample of 270 concussed patients (Fig. **2**).

### 3.2 Assessment of Demographic Variables on Outcomes

**Fig. (3):**
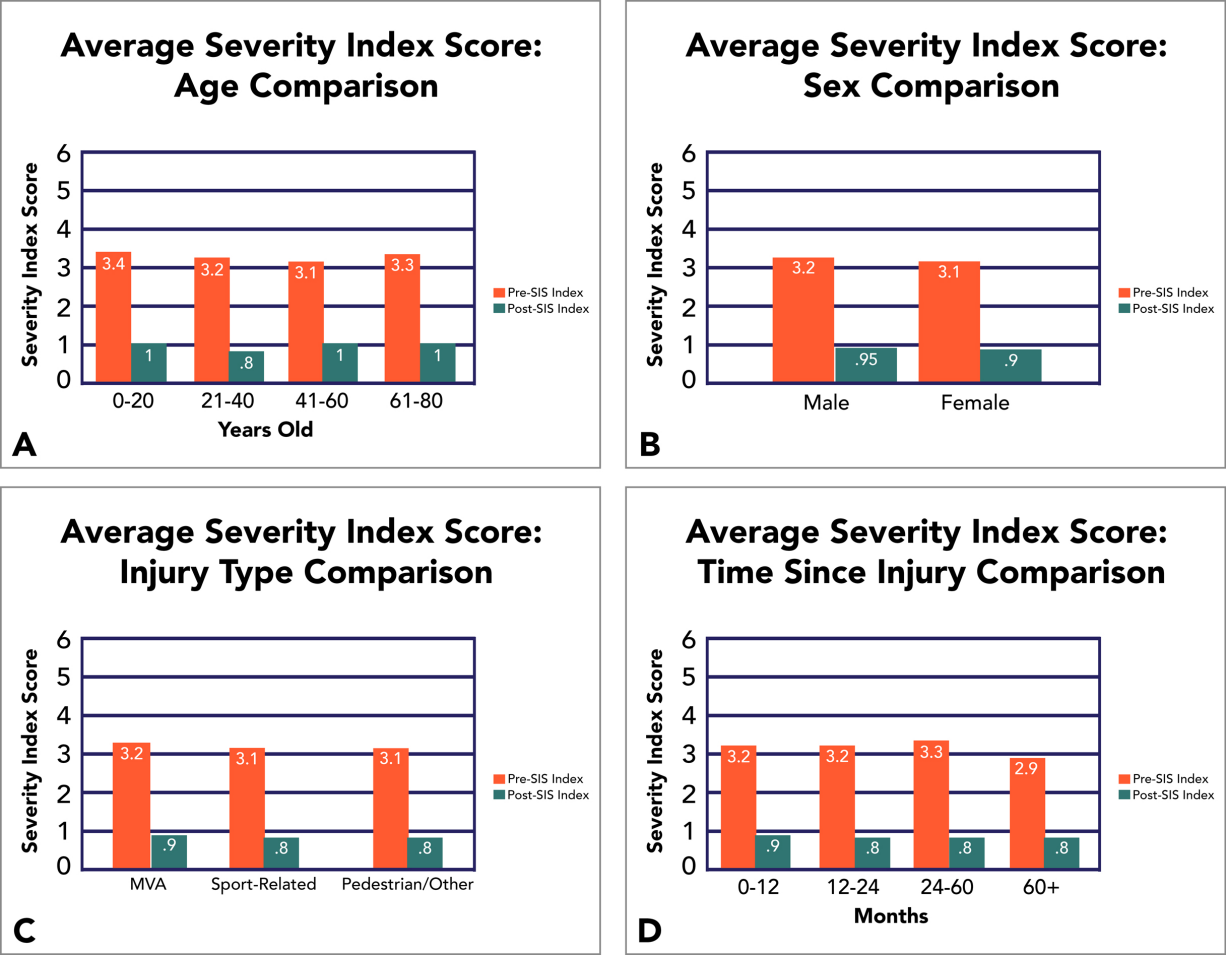
Averaged SIS Pre- and Post-Treatment with Comparisons for Demographic Variables. Improvement profiles appear largely unaffected by age (A), sex (B), mode-of-injury (C), and time since injury (D). MVA = Motor Vehicle Accident.

Considering a wide variety of concussion profiles with regards to age, sex, mode of injury, and time since injury, our EPIC treatment has also been shown to be extremely effective under both objective and subjective measures (Fig. **3**).

### 3.3 Improvement Outcomes

**Fig. (4):**
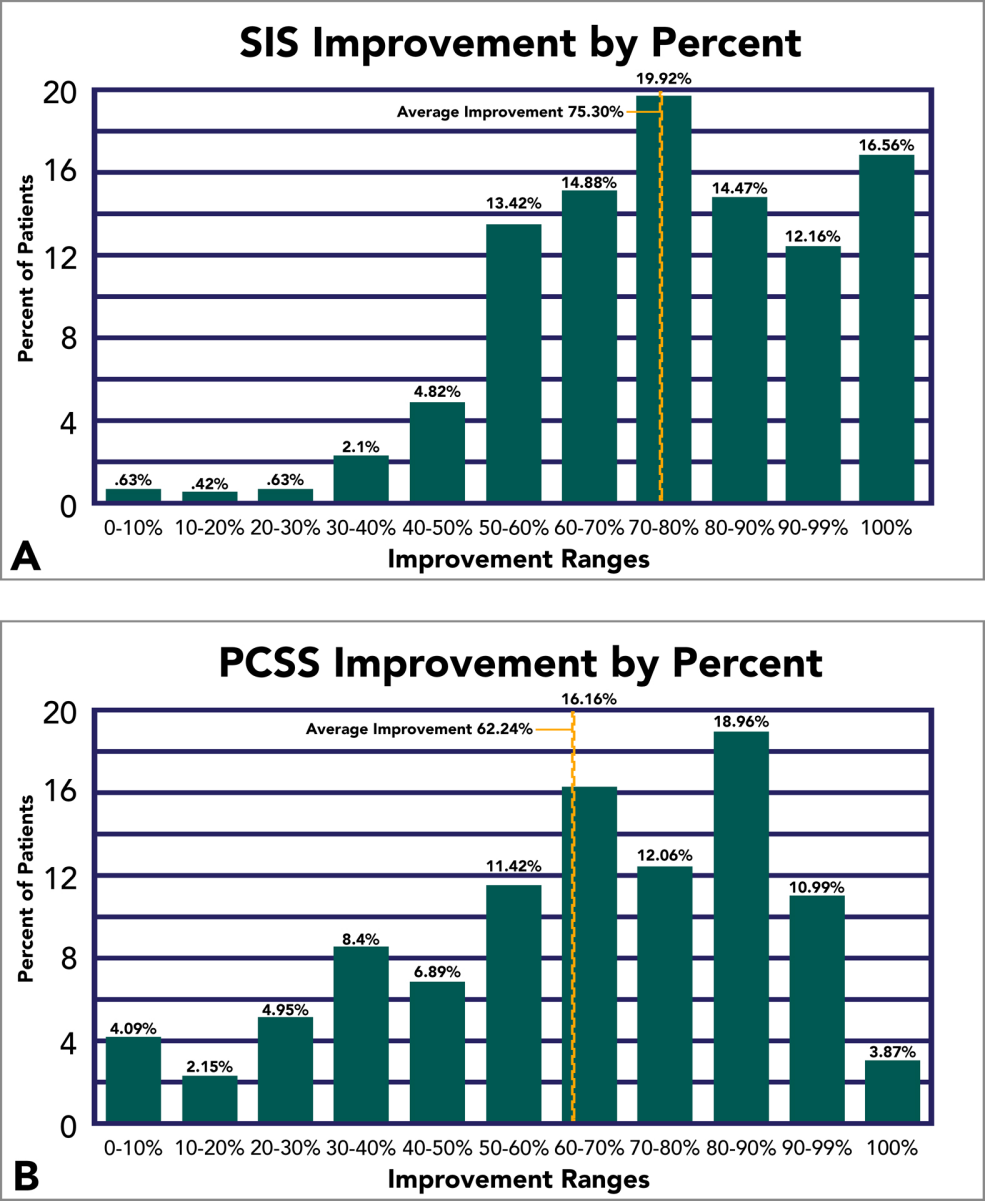
Improvement on SIS and PCSS Measurements. Average percent improvement is displayed for fNCI-based SIS outcomes (A), and PCSS outcomes (B). The percentage of patients (not cumulative) falling within each 10% improvement interval is displayed, as well as the percentage at 100% for both measures.

Improvement, in a broad sense, was been observed in 99.6% and 99.2% of our patients under objective and subject measurements, respectively. But the degree of improvement varied across a wide spectrum of percent differences (Range = 3.24 to 157.0%). Consequently, to address this complication, we established incremental improvement thresholds and report the total percentage of our patients that meet and/or exceed such thresholds. Average improvement is also graphically visualized (Fig. **4**).

### 3.4 Short-Term Outcome Comparison

**Fig. (5):**
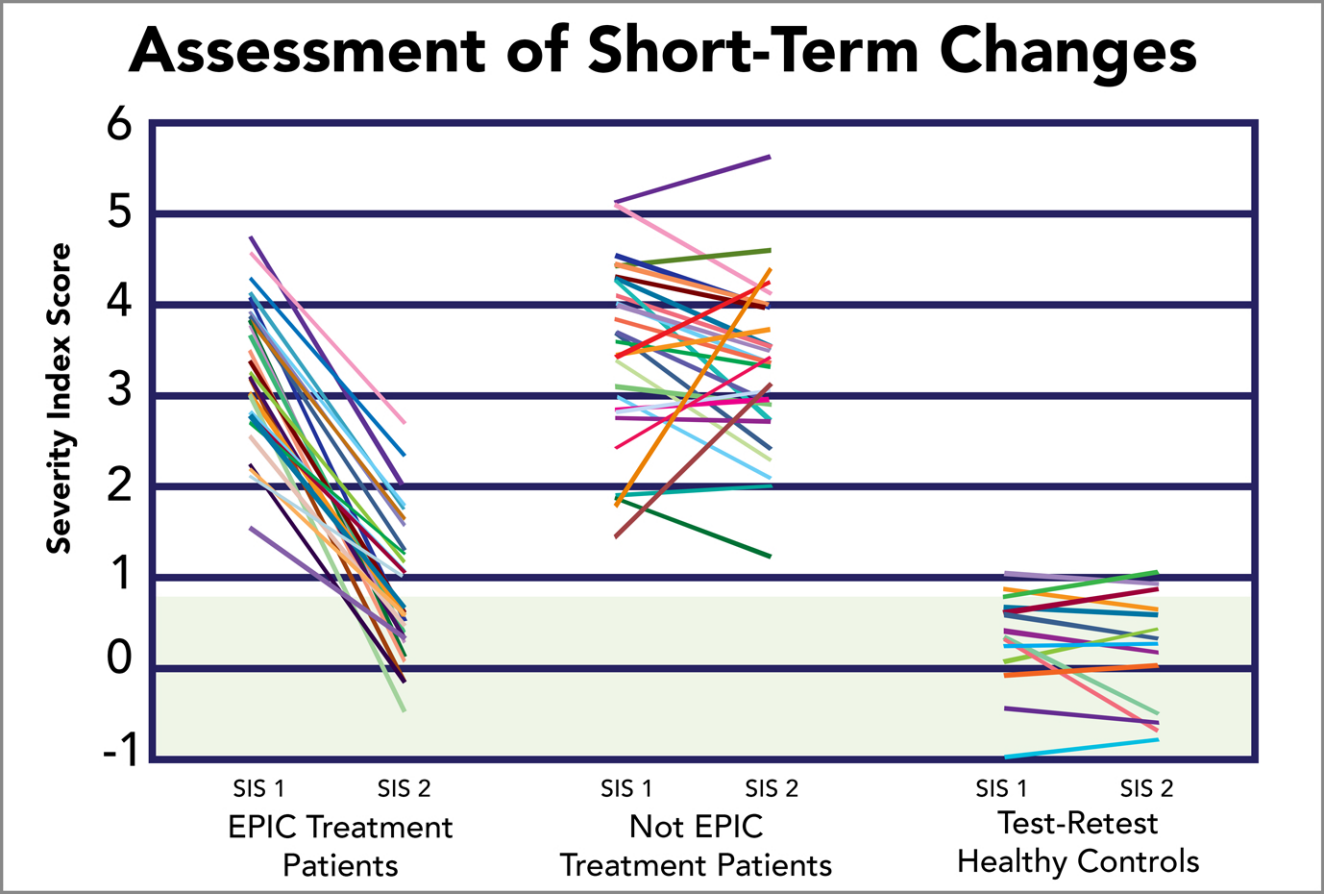
Assessment of Short-Term Changes on the SIS Measure. Random selection of patients who underwent EPIC treatment (1^st^ column), patients who elected not to participate in EPIC treatment (2^nd^ column) and healthy controls (3^rd^ column) were scanned at pre- and post-treatment time intervals. Green shading indicates normal SIS range.

A random sample of 27 patients who underwent EPIC treatment was generated to visually characterize the original pool of 270 patients. This sample was reported against a group of 27 patients who, at the time of initial scanning, opted to delay treatment and were rescanned at a later date. Additionally, it was comparatively reported against a sample of 13 healthy controls that were scanned in order to assess test-retest reliability of the fNCI method. The random sample accurately represents improvement trends reported above (Fig. **4**), observing improvement in 100% of patients, with an average of ~70% improvement amongst the treated patient sample (73.1% of which returned to within-normal-limits post-treatment. In comparison, 65.4% of patients who did not receive treatment saw spontaneous improvement, averaging 18.4% improvement (only 3.8% of which spontaneously returned to within-normal-limits) (Fig. **5**).

### 3.5 Longitudinal Outcomes

**Fig. (6):**
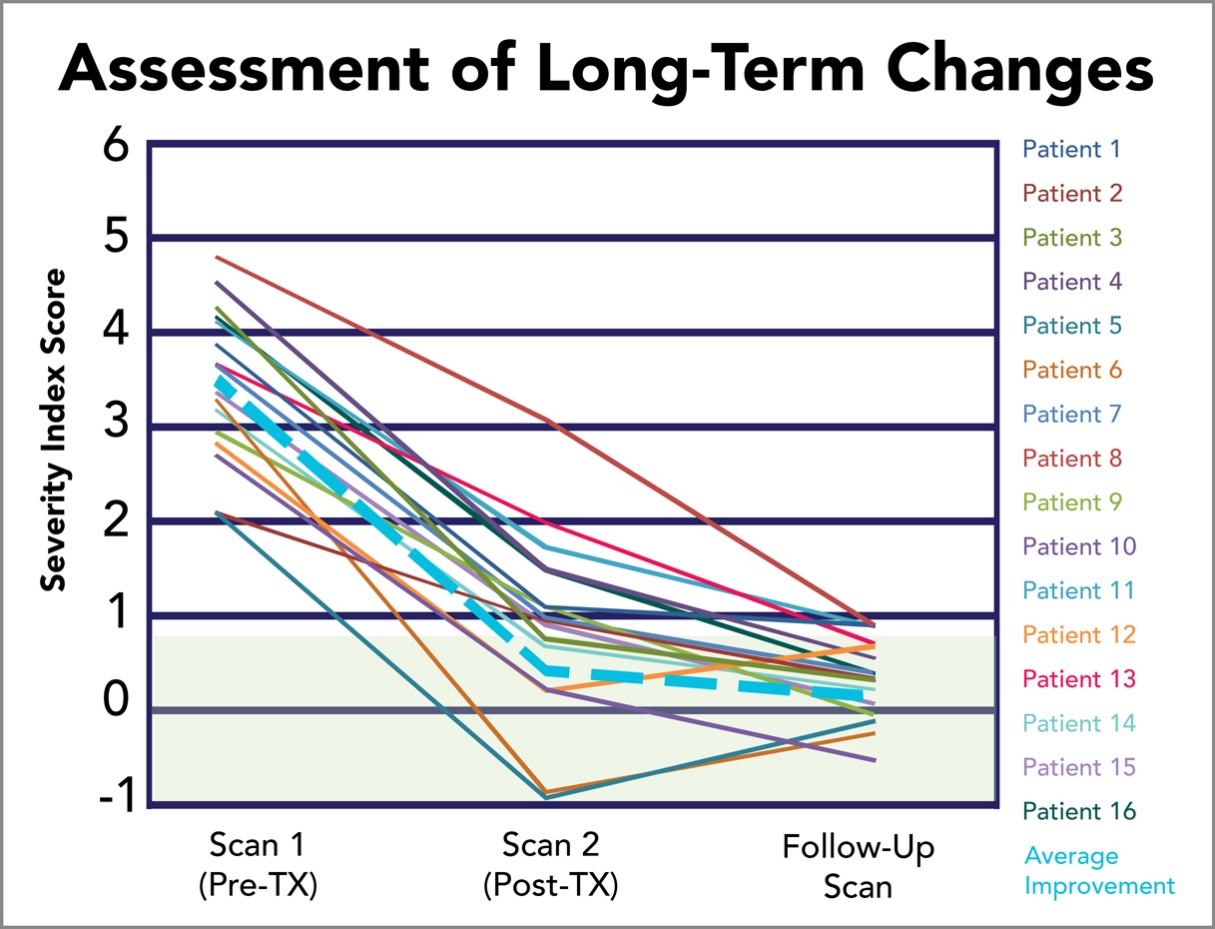
Assessment of Long-Term Changes. Sustained rehabilitative outcomes are observed for the SIS measure in longitudinal, follow-up scans averaging 8.8 months post-treatment. Green shading indicates normal SIS range.

Recovery in acute models of mTBI is often unpredictable. For those injuries that progress into persistent cases, research findings allude to the pathophysiological misalignment in default NVC efficiency. Accordingly, a rehabilitative approach that promotes the restoration of default NVC efficiency should, therefore, expect longevity of positive outcomes in PCS. As expected, longitudinal outcomes for fNCI-directed treatment report sustained rehabilitative outcomes in follow-up scans averaging 8.8 months post-treatment (Fig. **6**).

## 4 DISCUSSION

### 4.1 Clinical Function of Neurocognitive Imaging

Although traditional uses of fMRI and other advanced neuroimaging methods have been employed primarily for research purposes or preoperative functional mapping, we are rapidly approaching the standardized integration of these advanced imaging techniques in clinical evaluation [37, 38]. However, several key limitations have hindered the universal application and establishment of clinical fMRI practice. For the scope of this paper, we aim to present the two most general, yet major, limitations and briefly discuss our standardized approach to address their limiting effects on fNCI-specific data retrieval, contextualization, and clinical application.

In recent years, questions within the scientific community have been raised regarding the clinical reliability of fMRI because of rising skepticism towards BOLD signal stability. Skepticism revolves around the belief that fMRI data output (BOLD signal activation) is both unreliable and irreproducible [39, 40]. To provide a better framework that enforces BOLD signal stability, researchers have scientifically discovered a strong relationship between fMRI reliability characteristics and paradigm standardization [8, 40]. Through years of extensive pre-testing, *Notus NeuroCogs* methodology has been assessed for concurrent validity, reliability, objectivity, and suitability within the scanning environment using a standardized administration protocol [24-26, 41, 42]. In addition, our standardized functional task battery has been directly assessed for test-retest stability in a sample of 14 healthy controls to date, yielding a correlational average of r = 0.859 (StDev = 0.0904) amongst 57 task-associated regions (data specifics not shown).

The other well known, reoccurring argument made against clinical fMRI questions the appropriateness and sufficiency of statistical corrections made during fMRI data analysis [43]. Notably, all fMRI analyses include complex chains of statistics, but there is nowhere in our statistical pipeline that includes the type of inferential processes that would be subject to the concern of correcting for false positives. Avoiding these clinical criticisms associated with experimental methods of null hypothesis testing, our statistical contextualization approach removes concern through individualized analysis of raw patient data. Our strict adherence to raw data sidesteps the major issue surrounding inferential analyses (i.e. threshold-based significance testing), and places fNCI more in terms of fMRI’s objective implementation within its presurgical application. The cost of this clinically acceptable methodology, however, requires laborious work, employing a qualified neuroanatomist to assess both structural and functional variability unique to each brain, to ensure accurate identification of task-associated regions, thereby equating appropriate clinical relevance.

Using the same standardized administration presented to normative references, each patient was scanned using fNCI to establish pre-treatment benchmarks used in measuring ultimate therapeutic effectiveness. Activation data output was projected against the normative atlas to statistically contextualize degree of NVU present in any given brain region for each patient. Furthermore, regional NVU breakdown provided target regions of interest for neurorehabilitative efforts. This individualized localization of neurovascularly-uncoupled regions supports the clinical appropriateness of fNCI-directed treatment.

### 4.2 Biomarker Discovery

The normative reference atlas makes it possible to search for and verify biomarkers for specific pathologies [44, 45]—that is, reliable patterns of deviation from the norm associated with a specific pathology. Our PCS biomarker development followed a 3-step process: biomarker candidate search, independent samples validation, and multivariate base rate discovery.

#### 4.2.1 Biomarker Discovery: Biomarker Candidate Search

Biomarker candidate search was performed with a sample of 69 PCS patients (not included in the main study of this paper). As described above, the normative reference atlas identified 8-12 qualifying regions per each of the six fNCI exams for a total of 57 regions of stable activation in the initial sample of healthy control subjects. For the initial biomarker search, a *z*-score was computed (as a measure of deviation from the healthy control mean) for each of the 57 regions in 69 initial PCS patients. Prespecified functional-cognitive systems were targeted for analysis based on well-established fMRI experimental findings [46]. For example, the frontal attentional system (including medial prefrontal cortex and anterior insula) was one of 5 such targeted systems. Initial discovery criteria specified that activation must exceed and average of 2 SD (in either the hypo- or hyper-activation direction) for all regions belonging to the functional-cognitive system of interest (e.g., frontal attentional) across the six exams in at least 30% of patients. Five functional-cognitive systems met this criteria with initial sensitivity rates ranging from 40-90%. For example, the frontal attentional system was found consistently hypoactivated in > 80% of patients.

#### 4.2.2 Biomarker Discovery: Independent Samples Validation

Independent samples validation for true positive rates was performed with a new sample of 120 PCS patients. Resulting sensitivity calculations were consistent with the initial discovery finings to within +/- 5%. Assessment of false positive rates was performed using an additional sample of 52 healthy control subjects. Resulting specificity ranged from 96-100 for the 5 biomarkers. Thus, for example, the final sensitivity/specificity values for the frontal attentional biomarker (1 of the 5 biomarkers), following independent samples validation were 88/100, for that biomarker alone.

#### 4.2.3 Biomarker Discovery: Multivariate Base Rate Discovery

In order to address issues of false-positive bias with multiple biomarkers, a further multivariate base rate discovery procedure was carried out using additional samples of 70 PCS patients and 62 healthy control subjects, respectively. This method was adopted from Iverson and colleagues [47], in which diagnostic values are derived simultaneously from all 5 biomarkers. According to this analysis, using an appropriate statistical metric for multivariate false positive rates, the sensitivity/specificity values for all five biomarkers in aggregate are 88/99. However, for the purposes of computing SIS scores reported in this study, individual positive predictive values were computed for each biomarker using the standard sensitivity/specificity values in step 2 above, in order to provide individually weighted contributions from each biomarker as described above in section 2.3.

### 4.3 Positive Outcomes & Limitations

Longitudinal data collection has observed a maintained SIS within normal limits, signifying the probable restoration of pre-traumatic NVC and re-establishment of default NVC efficiency. Continued longitudinal data collection will likely explain the mechanistic observation of these positive outcomes.

At present, the dynamic approach of fNCI-directed EPIC treatment is unique with its intricate methodologies and implementation of multiple therapeutic elements. As noted previously, many reports demonstrate the effectiveness of physical, cognitive, and traditional rest independently in the rehabilitation of PCS [29-34, 48, 49]. And though a portion of these studies do monitor the effects of the combination of more than one therapeutic approach, no studies implement the three cyclical facets of EPIC treatment: Prepare, Activate, and Rest. Additionally, the accelerated timeframe and intensity of our program is unique when compared to these studies.

Due to the unique nature of our methodologies, one obvious limitation of our study is the ability to appropriately compare its effectiveness to other programs of similar treatment modalities. The lack of congruency in objective measurements fails to provide more accurate and thorough comparisons. However, when individually compared to exercise-, neurocognitive-, or rest-centered studies, EPIC treatment similarly reports improvement of acute symptomatology on subjective measurements [48-50]. But for more chronic cases such as PCS, the ability to target underlying pathology, as directed by fNCI, provides greater long-term symptom resolution due to the synergistic use of multiple therapeutic elements. Ideally, the methodologies employed by EPIC could be improved by having a “placebo treatment” condition in addition to the “no-treatment” group.

Though our positive treatment outcomes support the role of NVC in PCS, the complex interconnectedness of vast neural correlate systems found within the brain advocate the etiological potential of multiple mechanical failings (e.g. neuronal dysfunction, signaling/timing disruptions, and/or neurotransmitter dysregulation). Therefore, we support continual exploration of other advanced neurorehabilitative therapies and their relationships to persistent models of mTBI. However, standardized application of this evidence-based practice in general neurocognitive assessment and rehabilitation may be key to establishing clinical fMRI practice standards specifically in guiding neurorehabilitative outcomes.

## CONCLUSION

We present practice-based evidence for the standardized application of fNCI, which is currently used in directing neurorehabilitation, with particular efficiency in mTBI. We indicate the clinical appropriateness of this image-guided approach through iterative pilot testing, standardized administration, and individualized, normative-based contextualization. We further demonstrate the rehabilitative guidance capability of fNCI in establishing EPIC treatment standards that prioritize neurovascularly-uncoupled regions of interest unique to each patient, and support the targeted, multi-method, cyclical, and sustained approach employed by our multidisciplinary team. Positive patient outcomes—graphically represented using conventional and fNCI-directed measures—advocate fNCI’s efficacy in the accelerated and longitudinal neurorehabilitation of mTBI across a variety of concussion profiles.

## Figures and Tables

**Table 1:** Each of the six tasks within the *Notus NeuroCogs* battery have been listed with their respective objectives and operative descriptions.

***Notus NeuroCogs*^TM^**	
*Neuropsychologic Test*	*Task Description*
Functional Matrix Reasoning Test (*f-MRT*)	Tests non-verbal problem solving using a 3x3 array of visually complex figures with one figure missing. The subject is then instructed to select the best match for the missing figure from among four “candidate” figures by pressing a designated button
Functional Trail Making Test-B (*f-TMTB*)	Measures cognitive flexibility by presenting a virtual connect-the-dots tasks using a button pad response system. Randomly arranged numbers and letters are displayed on a screen and the subject must locate and connect each series of numbers and letters in ascending order while alternating back and forth between the two character types
Functional Picture Naming Test (*f-PNT*)	Assesses semantic object recognition by displaying line drawings of common objects for a period of 1.5 seconds each. Subjects are instructed to silently identify each object upon presentation
Functional Face Memory Test (*f-FMT*)	Investigates long-term memory. Subjects are instructed to memorize colored photographs of unfamiliar faces and informed that they will be required to identify some of the faces at a later time. Twenty faces are presented twice in 2 random orders for three seconds each during scanning. Recognition accuracy is recorded on a post-scan test
Functional Verbal Memory Test (*f-VMT*)	Analyzes short-term verbal memory. For each test run, the subject views a series of eight common words for one second each and is instructed to silently memorize the words as they appear. Subjects are given 12 additional seconds after all words have been presented to recall as many as possible
Functional Verbal Fluency Test (*f-VFT*)	A letter-based fluency test. The subject is instructed to silently generate as many unique words as possible (excluding proper names or variants of the same word) within a 20-second time limit using a given first letter

**Table 2:** Demographic details for normative reference volunteers.

**Normative Reference Volunteers (N = 59)**	
*Demographic*	*Breakdown*
Sex	Male (27): Female (32)
Handedness	Right-hand dominance (91.5%)
Ethnicity	Caucasian (74.6%); Hispanic (11.8%); Asian (10.2%); African American (3.4%)
First Language	English for all subjects
Years of School	At least one year of higher education for all subjects (Mean = 14.3, σ = 2.9)

**Table 3:** Patient demographic breakdowns are outlined.

**mTBI Patients (N = 270)**	
*Demographic*	*Breakdown*
Sex	Male (138): Female (132)
Age	Mean = 34.0, σ = 16.8 (years)
Handedness	Right-hand dominance (90.1%); Left-hand dominance (9.0%); Ambidextrous (0.9%)
Ethnicity	Caucasian (90.8%); Hispanic (5.5%); Asian (1.3%); Pacific Islander (0.9%); Native American (0.5%); Indian (0.5%); African American (0.5%)
First Language	English for all subjects
Years of School	Mean = 13.7, σ = 3.4
Mode of Injury	Motor Vehicle Accident (37.3%); Sport-related injury (35.5%); Pedestrian/Fall/Other (27.2%)
Inclusion Criteria	Any individual diagnosed with mTBI regardless of mode of or time since injury. All patients on initial trauma met criteria for mild-TBI and presented with an average Post-Concussion Symptom Scale index of 33.5 at the start of treatment.
Exclusion Criteria	Children under the age of 8 were excluded from this study. Additionally, any individual who could not result in conclusive data (e.g. due to braces or other metal disturbances)
